# Supplementary material for: Expanding the cytokine receptor alphabet reprograms T cells into diverse states
Source: Nature. 2025 Aug 13;645(8082):1039–50. doi: 10.1038/s41586-025-09393-1 (PMC12460165; doi:10.1038/s41586-025-09393-1)
Supplement: Supplementary file 1 — Flow cytometry gating strategy. This figure presents the gating strategy used to identify and analyse specific immune cell populations discussed in the main text. Gating steps were applied consistently across all samples, and representative plots are shown. Detailed gating steps and their correspondence to figures in the main text are described in the legend of Supplementary Fig. 1. [file 41586_2025_9393_MOESM1_ESM.pdf]

nature portfolio

https://doi.org/10.1038/s41586-025-09393-1

Supplementary information

Expanding the cytokine receptor alphabet reprograms T cells into diverse states

In the format provided by the authors and unedited

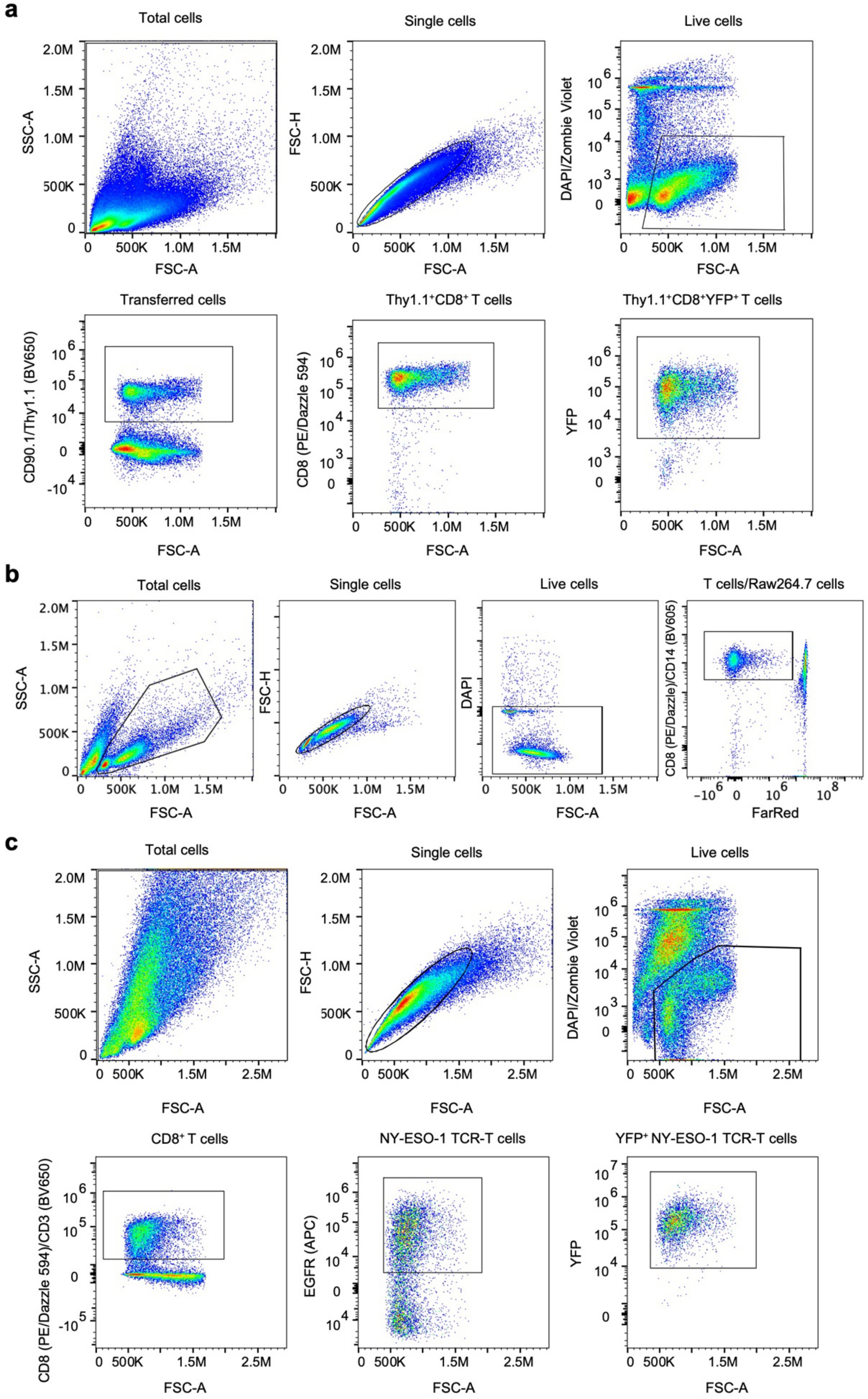
a
Total cells
SSC-A
FSC-A
Single cells
FSC-H
FSC-A
Live cells
DAPI/Zombie Violet
FSC-A
Transferred cells
CD90.1/Thy1.1 (BV650)
FSC-A
Thy1.1⁺CD8⁺ T cells
CD8 (PE/Dazzle 594)
FSC-A
Thy1.1⁺CD8⁺YFP⁺ T cells
YFP
FSC-A
b
Total cells
SSC-A
FSC-A
Single cells
FSC-H
FSC-A
Live cells
DAPI
FSC-A
T cells/Raw264.7 cells
CD8 (PE/Dazzle)/CD14 (BV605)
FarRed
c
Total cells
SSC-A
FSC-A
Single cells
FSC-H
FSC-A
Live cells
DAPI/Zombie Violet
FSC-A
CD8⁺ T cells
CD8 (PE/Dazzle 594)/CD3 (BV650)
FSC-A
NY-ESO-1 TCR-T cells
EGFR (APC)
FSC-A
YFP⁺ NY-ESO-1 TCR-T cells
YFP
FSC-A

d

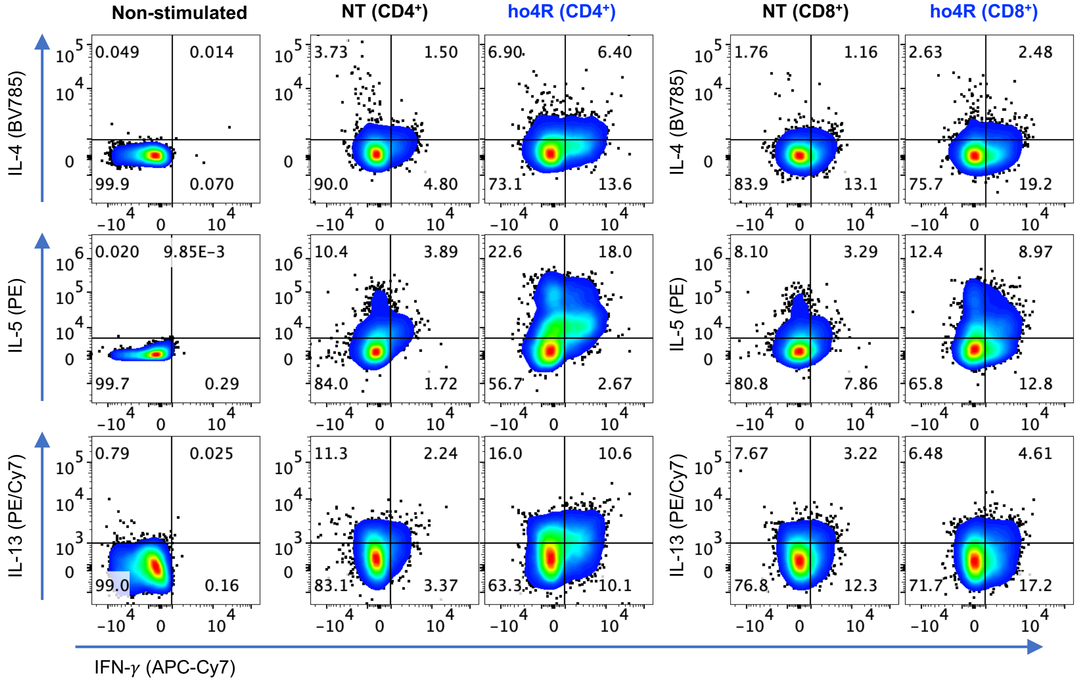

Supplementary Figure 1. Gating strategy.

a. Gating Strategy for in vivo pmel T cell phenotyping in Fig. 3j, Fig. 4g–l, Fig. 4p, Extended Data Fig. 4, and Extended Data Fig. 5h–k. Pmel T cells were gated using Thy1.1$^+$CD8$^+$, and orthogonal-receptor-expressing pmel T cells were gated using Thy1.1$^+$CD8$^+$YFP$^+$.

b. Gating Strategy for antibody-dependent cellular phagocytosis assay in Fig. 4n,o and Extended Data Fig. 6k. Phagocytic activity was measured as FarRed$^+$ cells within CD8$^+$ pmel T cells and CD14$^+$ RAW264.7 cells after gating for single, live cells and excluding FarRed$^+$ target cells.

c. Gating Strategy for in vivo NY-ESO-1 TCR-T cell phenotyping in Fig. 5k, Fig. 6h,i and Extended Data Fig. 11d. NY-ESO-1 TCR-T cells were gated using CD3$^+$EGFR$^+$, or CD8$^+$EGFR$^+$ for exhaustion marker and T_{SCM} analysis. Orthogonal-receptor-expressing NY-ESO-1 TCR-T cells were gated using CD3$^+$EGFR$^+$YFP$^+$, or CD8$^+$EGFR$^+$YFP$^+$ for exhaustion marker and T_{SCM} analysis. For the in vitro assays shown in Fig. 5c–h, Extended Data Fig. 7b,c,g, Extended Data Fig. 8e–j, Extended Data Fig. 10e,f, and Extended Data Fig. 11j–m, the same gating strategy was used to identify NY-ESO-1 TCR-T cells.

d. Gating Strategy for cytokine profiles of ho4R NY-ESO-1 TCR-T cells in Fig. 5c–g, and Extended Data Fig. 7b.
