# Supplementary material for: Expanding the cytokine receptor alphabet reprograms T cells into diverse states
Source: Nature. 2025 Aug 13;645(8082):1039–50. doi: 10.1038/s41586-025-09393-1 (PMC12460165; doi:10.1038/s41586-025-09393-1)
Supplement: Supplementary file 2 — Reporting Summary [file 41586_2025_9393_MOESM2_ESM.pdf]

nature portfolio

Corresponding author(s): K Christopher Garcia, Anusha Kalbasi

Last updated by author(s): Jun 17, 2025

Reporting Summary

Nature Portfolio wishes to improve the reproducibility of the work that we publish. This form provides structure for consistency and transparency in reporting. For further information on Nature Portfolio policies, see our Editorial Policies and the Editorial Policy Checklist.

Statistics

For all statistical analyses, confirm that the following items are present in the figure legend, table legend, main text, or Methods section.

n/a	Confirmed	
☐	☒	The exact sample size (n) for each experimental group/condition, given as a discrete number and unit of measurement
☐	☒	A statement on whether measurements were taken from distinct samples or whether the same sample was measured repeatedly
☐	☒	The statistical test(s) used AND whether they are one- or two-sided<br>*Only common tests should be described solely by name; describe more complex techniques in the Methods section.*
☐	☒	A description of all covariates tested
☐	☒	A description of any assumptions or corrections, such as tests of normality and adjustment for multiple comparisons
☐	☒	A full description of the statistical parameters including central tendency (e.g. means) or other basic estimates (e.g. regression coefficient) AND variation (e.g. standard deviation) or associated estimates of uncertainty (e.g. confidence intervals)
☐	☒	For null hypothesis testing, the test statistic (e.g. F, t, r) with confidence intervals, effect sizes, degrees of freedom and P value noted<br>*Give P values as exact values whenever suitable.*
☒	☐	For Bayesian analysis, information on the choice of priors and Markov chain Monte Carlo settings
☒	☐	For hierarchical and complex designs, identification of the appropriate level for tests and full reporting of outcomes
☒	☐	Estimates of effect sizes (e.g. Cohen's d, Pearson's r), indicating how they were calculated

Our web collection on statistics for biologists contains articles on many of the points above.

Software and code

Policy information about availability of computer code

Data collection	Flow cytometry data collection was performed with CytoFlex (Beckman Coulter). In vivo bioluminescence data collection was performed using a IVIS fluorescence/bioluminescence imaging system (Xenogen). Fluorescence imaging data collection was performed using a using a Elyra7 lattice SIM microscope (ZEISS) and Leica TCS SP8 Confocal with WLL. Bulk RNA sequencing data was collected on NovaSeq X Plus Series (PE150). scRNA sequencing data was collected on a NovaSeq 6000. Bulk ATAC sequencing data was collected using NovaSeqX sequencer. Incucyte data was collected on using the IncuCyte live imaging system (Essen Bioscience).
Data analysis	Flow cytometry data were analyzed using Flowjo (version 10.10.0). Statistical analysis was performed using Graphpad Prism (version 9). For bulk RNA sequencing, reads were aligned to the mouse reference genome (mm10) with Rsubread (version 2.18.0). Gene expression was quantified with featureCounts, and DESeq2 (version 1.48.1) was used for this downstream analysis. scRNA sequencing raw data were processed by cellranger mkfastq from 10x Genomics (version 7.1.0) using a custom reference package based on mouse reference genome mm10. The gene expression matrix was processed and analyzed using Seurat (version 5.1.0). Transcription factor (TF) activity prediction was conducted with the pySCENIC (version 0.12.1) docker distribution with default parameter settings. Intercellular communication prediction was performed using LIANA (version 1.5.1). Bulk ATAC sequencing were processed using RSubread (version 2.18.0) using reference genome (hg19). The aligned reads were subjected to peak calling with MACS3 (version 3.0.1). Downstream analyses were performed in R (version 4.4). Peaks were annotated with their genomic locations and associated genes using ChIPseeker (version 1.44.0). The reads aligned with each region were then counted with the summarizeOverlaps function, and the count matrix was analyzed with DESeq2 (version 1.48.1). Differential transcription factor (TF) motif enrichment analysis was performed using chromVAR (version 1.30.1). Cis-regulatory element enrichment analysis were performed with GREAT (version 2.10.0). The R code used to analyze the scRNA-seq data is publicly at Zenodo (DOI: 10.5281/zenodo.15702063). Analysis details are provided in the Methods section.

For manuscripts utilizing custom algorithms or software that are central to the research but not yet described in published literature, software must be made available to editors and reviewers. We strongly encourage code deposition in a community repository (e.g. GitHub). See the Nature Portfolio guidelines for submitting code & software for further information.

Data

Policy information about availability of data

All manuscripts must include a data availability statement. This statement should provide the following information, where applicable:

- Accession codes, unique identifiers, or web links for publicly available datasets
- A description of any restrictions on data availability
- For clinical datasets or third party data, please ensure that the statement adheres to our policy

The raw and processed bulk RNA-seq data are publicly available at GEO under accession code GSE282973 (C57BL/6 wild-type T cells; aligned to the mm10 mouse reference genome). The scRNA-seq data are available at GSE272444 (TCR transgenic pmel T cells in melanoma tumors; mm10 genome). ATAC-seq data generated in this study have been deposited at GEO under accession code GSE272385 (human T cells in melanoma tumors grown in NSG mice; aligned to the hg19 human reference genome). Normalized gene expression matrices and associated sample metadata were downloaded from the Gene Expression Omnibus (https://www.ncbi.nlm.nih.gov/geo/) using the accession numbers above. All other raw data are available on Figshare (DOI: 10.6084/m9.figshare.26322181). Additional supporting information is available from the corresponding author upon reasonable request.

Research involving human participants, their data, or biological material

Policy information about studies with human participants or human data. See also policy information about sex, gender (identity/presentation), and sexual orientation and race, ethnicity and racism.

Reporting on sex and gender	Gender information was not collected.
Reporting on race, ethnicity, or other socially relevant groupings	N/A
Population characteristics	Human T cells were isolated from buffy coats from anonymous healthy donors (male and female; gender information was not collected) purchased from the Stanford Blood Center.
Recruitment	Anonymous healthy donors were recruited by the Stanford Blood Center.
Ethics oversight	Ethical approval pertaining to T cell donors was obtained by the Stanford Blood Center.

Note that full information on the approval of the study protocol must also be provided in the manuscript.

Field-specific reporting

Please select the one below that is the best fit for your research. If you are not sure, read the appropriate sections before making your selection.

☒ Life sciences ☐ Behavioural & social sciences ☐ Ecological, evolutionary & environmental sciences

For a reference copy of the document with all sections, see nature.com/documents/nr-reporting-summary-flat.pdf

Life sciences study design

All studies must disclose on these points even when the disclosure is negative.

Sample size	Group sizes for in vivo validation experiments were selected empirically based on previous results of the intragroup variation of tumor growth upon similar treatments. Similarly, group sizes in vitro were selected on the basis of prior knowledge of variation. Ref: Nature Biotechnology volume 42, pages1693–1704 (2024). doi: 10.1038/s41587-023-02060-8.
Data exclusions	Rout outlier tests were run with default parameters (Q = 1%) in Prism on all mouse experimental data due to inherent variability within the model system.
Replication	All presented results were repeatable. Replicates were used in all experiments as noted in figure captions or methods.
Randomization	Age and sex-matched animals were used for each experiment. Mice were randomized prior to treatment. In the in vitro experiments, samples with same pretreatment conditions were randomly assigned to a treatment group.
Blinding	No blinding was performed due to requirements for cage labeling and staffing needs.

Reporting for specific materials, systems and methods

We require information from authors about some types of materials, experimental systems and methods used in many studies. Here, indicate whether each material, system or method listed is relevant to your study. If you are not sure if a list item applies to your research, read the appropriate section before selecting a response.

Materials & experimental systems

n/a	Involved in the study
☐	☒ Antibodies
☐	☒ Eukaryotic cell lines
☒	☐ Palaeontology and archaeology
☐	☒ Animals and other organisms
☒	☐ Clinical data
☒	☐ Dual use research of concern
☒	☐ Plants

Methods

n/a	Involved in the study
☒	☐ ChIP-seq
☐	☒ Flow cytometry
☒	☐ MRI-based neuroimaging

Antibodies

Antibodies used

The following antibodies or staining reagents were purchased from BioLegend: mouse CD16/32 (93, 101302), mouse CD90.1/Thy-1.1 (OX-7, 202533), mouse CD8b (YTS156.7.7, 126606), mouse CD8b (YTS156.7.7, 126614), mouse CD45.2 (104, 109808), mouse Ki-67 (16A8, 652413), mouse SCA-1 (D7,108142), mouse CD44 (IM7, 103030), mouse CD62L (MEL-14, 104428), mouse PD-1 (29F.1A12, 135220), mouse IFNγ (XMG1.2, 505850), mouse TNF-α (MP6-XT22, 506329), mouse/human Granzyme B (QA16A02, 372214), mouse/human KLRG1 (2F1/KLRG1, 138419), mouse IL-7Rα (A7R34, 135022), mouse CD122 (TM-β1, 123210), mouse SLAMF6 (330-AJ, 134606), mouse CD11b (M1/70, 101228), mouse CD14 (Sa14-2, 123335), mouse CD64 (X54-5/7.1, 139303), mouse LY6G (1A8, 127648), mouse SIRPα (P84, 144011), human EGFR (AY13, 352906), human CD3 (HIT3a, 300308), human CD4 (SK3, 344646), human CD8 (SK1, 344724), human IL-13 (JES10-5A2, 501914), human IL-4 (MP4-25D2, 500845), human IL-5 (TRFK5, 504304), mouse/human GATA3 (W19195B, 386906), human CXCR3 (G025H7, 353737), human CCR4 (L291H4, 359443), human CD62L (DREG-56, 304830), human CD95 (DX2, 305622), human CD45RA (HI100, 304120), human CD27 (O323, 302832), human CCR7 (G043H7, 353214), human CD45RO (UCHL1, 304228), human CD66b (G10F5, 305121), human LAG-3 (11C3C65, 369322), human IFNγ (4S.B3, 502530), human Ki-67 (Ki-67, 350526), human CD39 (A1, 328240), human TIM-3 (F38-2E2, 345026), mouse CD64 (X54-5/7.1, 139301), Human TruStain FcX™ (422302), anti-mouse CD3ε (145-2C11) and Zombie Violet™ Fixable Viability Kit (423114). The following antibodies or staining reagents were purchased from BD Biosciences: pSTAT3 (4/P-STAT3, 557815), pSTAT4 (38/p-Stat4, 558137), pSTAT5 (47/Stat5, 612599), mouse pSTAT6 (J71-773.58.11, 558252), human pSTAT6 (23/Stat6, 612701), and BD Pharmingen™ APC BrdU Kit (552598). The following antibodies were purchased from Cell Signal: pSTAT1 (58D6, 8009S) and Myc-Tag (9B11, 3739/2233S). The following antibodies were purchased from BioXcell: anti-mouse CD16/32 (93), Anti-human IL-4 (MP4-25D2), Anti-human IL-5 (TRFK5), Anti-human IL-13 (Tralokinumab), anti-mouse CD28 (37.51), anti-human CD3ε (OKT-3) and anti-human CD28 (9.3).For flow cytometry staining, surface marker antibodies were used at a 1:200 dilution, intracellular antibodies at 1:100, and pSTAT antibodies at 1:50.

Validation

Here are the links of the websites that show validation of the antibodies used in this study:
mouse CD16/32 (93, 101302): https://www.biolegend.com/en-us/search-results/apc-anti-mouse-cd16-32-antibody-6282
mouse CD90.1/Thy-1.1 (OX-7, 202533): https://www.biolegend.com/en-us/products/apc-anti-rat-cd90-mouse-cd901-thy11-antibody-5621
mouse CD8b (YTS156.7.7, 126606): https://punchout.biolegend.com/en-us/products/fitc-anti-mouse-cd8b-antibody-4475
mouse CD8b (YTS156.7.7, 126614): https://www.biolegend.com/en-us/products/apc-anti-mouse-cd8b-antibody-9055
mouse CD45.2 (104, 109808): https://www.biolegend.com/en-us/products/pe-anti-mouse-cd45-2-antibody-7?GroupID=BLG7007
mouse Ki-67 (16A8, 652413): https://www.biolegend.com/en-us/products/brilliant-violet-605-anti-mouse-ki-67-antibody-8983
mouse SCA-1 (D7,108142): https://www.biolegend.com/en-us/products/alexa-fluor-700-anti-mouse-ly-6a-e-sca-1-antibody-12078
mouse CD44 (IM7, 103030): https://www.biolegend.com/en-us/products/pe-cyanine7-anti-mouse-human-cd44-antibody-3932
mouse CD62L (MEL-14, 104428): https://www.biolegend.com/en-us/search-results/apc-anti-mouse-cd62l-antibody-381
mouse PD-1 (29F.1A12, 135220): https://www.biolegend.com/en-us/products/brilliant-violet-605-anti-mouse-cd279-pd-1-antibody-7648?GroupID=BLG7927
mouse IFNγ (XMG1.2, 505850): https://www.biolegend.com/en-us/products/apc-cyanine7-anti-mouse-ifn-gamma-antibody-13155
mouse TNF-α (MP6-XT22, 506329): https://www.biolegend.com/en-us/products/brilliant-violet-605-anti-mouse-tnf-alpha-antibody-7682
mouse/human Granzyme B (QA16A02, 372214): https://www.biolegend.com/en-us/products/pe-cyanine7-anti-humanmouse-granzyme-b-recombinant-antibody-15582
mouse/human KLRG1 (2F1/KLRG1, 138419): https://www.biolegend.com/en-us/products/brilliant-violet-605-anti-mouse-human-klrg1-mafa-antibody-9644
mouse IL-7Rα (A7R34, 135022): https://www.biolegend.com/en-us/products/percp-cyanine5-5-anti-mouse-cd127-il-7ralpha-antibody-6196
mouse CD122 (TM-β1, 123210): https://www.biolegend.com/en-us/products/pe-anti-mouse-cd122-il-2rbeta-antibody-4160
mouse SLAMF6 (330-AJ, 134606): https://www.biolegend.com/en-us/products/pe-anti-mouse-ly108-antibody-6016
mouse CD11b (M1/70, 101228): https://www.biolegend.com/en-us/products/percp-cyanine5-5-anti-mouse-human-cd11b-antibody-4257
mouse CD14 (Sa14-2, 123335): https://www.biolegend.com/en-us/products/brilliant-violet-605-anti-mouse-cd14-antibody-20001
mouse CD64 (X54-5/7.1, 139303): https://www.biolegend.com/en-us/products/pe-anti-mouse-cd64-fcgammari-antibody-6691
mouse LY6G (1A8, 127648): https://www.biolegend.com/en-us/products/pe-dazzle-594-anti-mouse-ly-6g-antibody-12246
mouse SIRPα (P84, 144011): https://www.biolegend.com/en-us/products/pe-anti-mouse-cd172a-sirpalpha-antibody-9801
human EGFR (AY13, 352906): https://www.biolegend.com/en-us/products/apc-anti-human-egfr-antibody-7714
human CD3 (HIT3a, 300308): https://www.biolegend.com/en-us/products/pe-anti-human-cd3-antibody-753
human CD4 (SK3, 344646): https://www.biolegend.com/en-us/products/brilliant-violet-605-anti-human-cd4-antibody-15992?GroupID=GROUP28
human CD8 (SK1, 344724): https://www.biolegend.com/en-us/products/alexa-fluor-700-anti-human-cd8-antibody-9062
human IL-13 (JES10-5A2, 501914): https://www.biolegend.com/en-us/products/pe-cyanine7-anti-human-il-13-antibody-13129
human IL-4 (MP4-25D2, 500845): https://www.biolegend.com/en-us/products/brilliant-violet-785-anti-human-il-4-antibody-24058

human IL-5 (TRFK5, 504304): https://www.biolegend.com/en-us/products/pe-anti-mouse-human-il-5-antibody-991
mouse/human GATA3 (W19195B, 386906): https://www.biolegend.com/en-us/products/apc-fire-750-anti-gata3-antibody-24357
human CXCR3 (G025H7, 353737): https://www.biolegend.com/en-us/products/brilliant-violet-785-anti-human-cd183-cxcr3-antibody-12124
human CCR4 (L291H4, 359443):https://www.biolegend.com/en-us/products/alexa-fluor-700-anti-human-cd194-ccr4-antibody-24167
human CD62L (DREG-56, 304830): https://www.biolegend.com/en-us/products/brilliant-violet-785-anti-human-cd62l-antibody-7974
human CD95 (DX2, 305622): https://www.biolegend.com/en-us/products/pe-cyanine7-anti-human-cd95-fas-antibody-6495
human CD45RA (HI100, 304120): https://www.biolegend.com/en-us/products/alexa-fluor-700-anti-human-cd45ra-antibody-3421
human CD27 (O323, 302832): https://www.biolegend.com/en-us/products/brilliant-violet-785-anti-human-cd27-antibody-7970
human CCR7 (G043H7, 353214): https://www.biolegend.com/en-us/products/apc-anti-human-cd197-ccr7-antibody-7536
human CD45RO (UCHL1, 304228): https://www.biolegend.com/en-us/products/apc-cyanine7-anti-human-cd45ro-antibody-7372
human CD66b (G10F5, 305121): https://www.biolegend.com/en-us/products/pe-dazzle-594-anti-human-cd66b-antibody-13979
human LAG-3 (11C3C65, 369322): https://www.biolegend.com/en-us/products/brilliant-violet-785-anti-human-cd223-lag-3-antibody-14877
human IFNγ (4S.B3, 502530): https://www.biolegend.com/en-us/products/apc-cyanine7-anti-human-ifn-gamma-antibody-6965
human Ki-67 (Ki-67, 350526): https://www.biolegend.com/en-us/products/pe-cyanine7-anti-human-ki-67-antibody-9084
human CD39 (A1, 328240): https://www.biolegend.com/en-us/products/brilliant-violet-785-anti-human-cd39-antibody-18339
human TIM-3 (F38-2E2, 345026): https://www.biolegend.com/en-us/products/apc-cyanine7-anti-human-cd366-tim-3-antibody-11928
mouse CD64 (X54-5/7.1, 139301): https://www.biolegend.com/en-gb/products/purified-anti-mouse-cd64-fcgammari-antibody-6690?GroupID=BLG8810
Human TruStain FcX™ (422302): https://www.biolegend.com/en-us/products/human-trustain-fcx-fc-receptor-blocking-solution-6462
anti-mouse CD3ε (145-2C11): https://www.biolegend.com/en-us/products/purified-anti-mouse-cd3epsilon-antibody-28
Zombie Violet™ Fixable Viability Kit (423114): https://www.biolegend.com/en-us/products/zombie-violet-fixable-viability-kit-9341
pSTAT3 (4/P-STAT3, 557815): https://www.bdbiosciences.com/en-us/products/reagents/flow-cytometry-reagents/research-reagents/single-color-antibodies-ruo/alexa-fluor-647-mouse-anti-stat3-py705.557815
pSTAT4 (38/p-Stat4, 558137): https://www.bdbiosciences.com/en-us/products/reagents/flow-cytometry-reagents/research-reagents/single-color-antibodies-ruo/alexa-fluor-647-mouse-anti-stat4-py693.558137
pSTAT5 (47/Stat5, 612599): https://www.bdbiosciences.com/en-us/products/reagents/flow-cytometry-reagents/research-reagents/single-color-antibodies-ruo/alexa-fluor-647-mouse-anti-stat5-py694.612599
mouse pSTAT6 (J71-773.58.11, 558252): https://www.bdbiosciences.com/en-us/products/reagents/flow-cytometry-reagents/research-reagents/single-color-antibodies-ruo/pe-mouse-anti-mouse-stat6-py641.558252
human pSTAT6 (23/Stat6, 612701): https://www.bdbiosciences.com/en-us/products/reagents/flow-cytometry-reagents/research-reagents/single-color-antibodies-ruo/pe-mouse-anti-stat6-py641.612701
BD Pharmingen™ APC BrdU Kit (552598): https://www.bdbiosciences.com/en-us/products/reagents/flow-cytometry-reagents/research-reagents/cell-function-analysis-stains-dyes/apc-brdu-kit.552598
pSTAT1 (58D6, 8009S): https://www.cellsignal.com/products/antibody-conjugates/phospho-stat1-tyr701-58d6-rabbit-mab-alexa-fluor-647-conjugate/8009
Myc-Tag (9B11, 3739/2233S): https://www.cellsignal.com/products/antibody-conjugates/myc-tag-9b11-mouse-mab-pe-conjugate/3739
Anti-human IL-4 (MP4-25D2): https://bioxcell.com/invivomab-anti-human-il-4-be0240
Anti-human IL-5 (TRFK5): https://bioxcell.com/invivomab-anti-mouse-human-il-5-be0198
Anti-human IL-13 (Tralokinumab): https://bioxcell.com/invivosim-anti-human-il-13-tralokinumab-biosimilar-sim0042
anti-mouse CD28 (37.51): https://bioxcell.com/invivomab-anti-mouse-cd28-be0015-1
anti-human CD3ε (OKT-3): https://bioxcell.com/invivomab-anti-human-cd3-be0001-2
anti-human CD28 (9.3): https://bioxcell.com/invivomab-anti-human-cd28-be0248

Eukaryotic cell lines

Policy information about cell lines and Sex and Gender in Research

Cell line source(s)

The B16F10 mouse melanoma cell line, HEK293T cell, Raji cell, RAW 264.7 cell, and J774A.1 cell were originally acquired from the American Type Culture Collection (ATCC). Platinum-E (Plat-E) and Platinum-GP (Plat-GP) retroviral Packaging Cell Line were purchased from Cell Biolabs. A375 and nRFP-M407 human melanoma cells were provided by Prof. Antoni Ribas (University of California Los Angeles). Mouse B cell lymphoma A20 cells were provided by Prof. Robert S. Negrin (Stanford University) and originally sourced from ATCC. The NALM6 cell line was obtained from ATCC. NALM6 cells were modified to express firefly luciferase (NALM6-Luc).

Authentication

None of the cell lines were authenticated in these studies. In all studies, cell lines with low passage number were used.

Mycoplasma contamination

All cell lines were confirmed mycoplasma negative.

Commonly misidentified lines
(See ICLAC register)

No commonly misidentified cell lines were used.

Animals and other research organisms

Policy information about studies involving animals; ARRIVE guidelines recommended for reporting animal research, and Sex and Gender in Research

Laboratory animals

Five- to six-week-old female Thy1.2+ C57BL/6 (C57BL/6J) mice and five- to seven-week-old NOD.Cg-Prkdcscid Il2rgtm1Wjl/SzJ (NSG) mice were purchased from Jackson Laboratory. Five- to six-week-old TCR-transgenic Thy1.1+ pmel-1 (pmel) mice (B6.Cg-Thy1a/Cy Tg(TcraTcrb)8Rest/J) were originally purchased from the Jackson Laboratory and maintained in the Stanford University-Lorry Lokey

(SIM1) Facility.

Wild animals: Study did not involve wild animals.

Reporting on sex: Female mice were used for all the experiments.

Field-collected samples: Study id not involve field-collected samples.

Ethics oversight: Mice were housed in animal facilities approved by the Association for the Assessment and Accreditation of Laboratory Care. Experimental procedures in mouse studies were approved by the Institutional Animal Care and Use Committee (IACUC) at the Stanford University (animal protocol ID 32279) and performed in accordance with the guidelines from the animal facility of Stanford University.

Note that full information on the approval of the study protocol must also be provided in the manuscript.

Plants

Seed stocks: N/A

Novel plant genotypes: N/A

Authentication: N/A

Flow Cytometry

Plots

Confirm that:

☒ The axis labels state the marker and fluorochrome used (e.g. CD4-FITC).

☒ The axis scales are clearly visible. Include numbers along axes only for bottom left plot of group (a 'group' is an analysis of identical markers).

☒ All plots are contour plots with outliers or pseudocolor plots.

☒ A numerical value for number of cells or percentage (with statistics) is provided.

Methodology

Sample preparation: For in vivo samples, collected tumors were weighed, mechanically minced, and digested in RPMI-1640 medium supplemented with collagenase type IV (1 mg/ml, Gibco/Thermo Fisher Scientific), dispase II (100 μg ml−1, Sigma-Aldrich), hyalurondase (100 μg ml−1, Sigma-Aldrich), and DNase I (100 μg ml−1, Sigma-Aldrich) at 37 °C for 60 min. RBC lysis was performed on the digested tumor samples with ACK lysing buffer. Tumor infiltrating leukocytes were then enriched by Percoll (Cytiva) density gradient centrifugation, resuspended in PBS with BSA (0.2%, wt/v), stained with indicated antibodies, and analyzed by flow cytometry. Spleens were ground and filtered through a 70-μm strainer (Fisher Scientific). RBC lysis was performed on the spleen samples with ACK lysing buffer (2 ml per spleen, Gibco/Thermo Fisher Scientific) and then resuspended in PBS with BSA (0.2%, wt/v). TDLNs were ground and filtered through a 70-μm strainer (Fisher Scientific) and then resuspended in PBS with BSA (0.2%, wt/v).

Instrument: CytoFlex (Beckman Coulter)

Software: FlowJo (v 10.10.0)

Cell population abundance: Among the enriched live singlet Tumor-Infiltrating Lymphocytes, the proportion of transferred T cells ranges from 0.4% to 5.0%

Gating strategy: Pmel CD8+ T cells were gated based on Thy1.1+CD8+, and orthogonal chimeric receptor-engineered pmel CD8+ cells were gated using Thy1.1+YFP+CD8+. For antibody-dependent cellular phagocytosis assays, phagocytic activity was measured as FarRed+ cells within CD8+ pmel T cells and CD14+ RAW264.7 cells after gating for single, live cells and excluding target cells. CD19 CAR T cells were gated based on CD3+Myc+, and orthogonal chimeric receptor-engineered CD19 CAR cells were gated using CD3+YFP+Myc+. NY-ESO-1 TCR-T cells were gated based on CD3+EGFR+, and orthogonal chimeric receptor-engineered NY-ESO-1 TCR-T cells were gated using CD3+YFP+EGFR+.

☒ Tick this box to confirm that a figure exemplifying the gating strategy is provided in the Supplementary Information.
